# An epidemiological study estimating the burden of cancer risk in patients with Raynaud’s phenomenon

**DOI:** 10.1007/s10067-026-07961-y

**Published:** 2026-01-29

**Authors:** Michael Hughes, Edward Jude, Zsuzsanna H. McMahan, Uazman Alam, Barbara Ruaro, Sizheng Steven Zhao

**Affiliations:** 1 Centre for Musculoskeletal Research, Division of Musculoskeletal and Dermatological Science, School of Biological Sciences, Faculty of Biological Medicine and Health, The University of Manchester, Manchester Academic Health Science Centre, Stopford Building, Oxford Road, Manchester, UK; 2 Department of Rheumatology, Northern Care Alliance NHS Foundation Trust, Salford Care Organisation, Salford, UK; 3 NIHR Manchester Biomedical Research Centre, Manchester University NHS Foundation Trust, Manchester, UK; 4 Tameside and Glossop Integrated Care NHS Foundation Trust, Ashton under Lyne, UK; 5 University of Manchester, Manchester, UK; 6 Manchester Metropolitan University, Manchester, UK; 7 Department of Medicine, Division of Rheumatology, UTHealth Houston, Houston, TX USA; 8 Institute of Life Course and Medical Sciences, University of Liverpool, Liverpool, UK; 9 Department of Medicine, University Hospital Aintree, Liverpool University NHS Foundation Trust, Liverpool, UK; 10 Department of Medical Surgical and Health Sciences, Hospital of Cattinara, University of Trieste, Pulmonology Unit, Trieste, Italy

**Keywords:** Autoimmunity, Cancer, Malignancy, Raynaud’s phenomenon, Rheumatology, Systemic sclerosis

## Abstract

**Introduction/Objective:**

Raynaud’s phenomenon (RP) is a common vasospastic condition that may develop secondary to cancer and/or in association with systemic autoimmune rheumatic diseases (SARDs). We aimed to estimate the risk of cancer in RP without known SARDs, phenotypically akin to ‘primary’ RP.

**Methods:**

A cohort study using data from North American electronic healthcare organization records. RP was defined using ≥ 2 ICD (I73.0) codes, excluding SARDs. Comparators had ≥ 2 irritable bowel syndrome ICD (K58) codes: selected with similar epidemiology to RP, and without any known excess in cancer risk. Cohorts were stratified by age (< 45 and ≥ 45 years). Our primary outcome was any cancer event. Secondary outcomes were rates of specific cancers: head and neck, digestive, thorax, skin, breast, haematological, male/female genital. Risk of each outcome was compared using 1:1 propensity score-matched Cox proportional hazard models.

**Results:**

Among 34,582 (< 45 years) and 68,836 (≥ 45 years) matched pairs, the hazard ratio (HR) of any cancer was higher in RP: < 45 [1.11 (1.03, 1.19)] and ≥ 45 [1.08 (1.05, 1.11)]. Cancer-specific risks were calculated. RP was associated in both age groups with increased risk of thorax (HR 2.077 & 1.433), skin (HR 1.202 &1.213), and haematological (HR 1.647 & 1.338) cancers. RP was associated with decreased risk of digestive cancer (HR 0.686 & 0.894).

**Conclusion:**

RP was associated with an increased risk of cancer, independent of age. We also describe varying cancer-specific risks. Future research is warranted to confirm and explore these novel observations, including potentially shared pathobiological mechanisms.

**Table:** 

**Key Points** • *RP was associated with an overall increased risk of all cancers in younger (<45years) and older (≥45 years) individuals.* • *There was an increased risk of certain (e.g., thorax, skin, and haematological) cancers with RP.* • *A reduced risk of cancer was also noted for certain cancers, especially GI-related.* • *Future research should confirm and explore these observations, including the pathobiology underpinning potential cancer risk in RP.*

**Supplementary Information:**

The online version contains supplementary material available at 10.1007/s10067-026-07961-y.

## Introduction

Raynaud’s phenomenon (RP) is a common condition characterised by episodic painful digital vasospasm, affecting ~ 3–5% of the general population [1–3]. However, higher population estimates of RP have been reported in some community-based studies in men (4–14%) and women (5–20%) [4, 5]. The majority of (80–90%) individuals have primary RP (PRP), which typically occurs in younger females, with the onset often occurring before 30 years of age [6]. Significant progress has been made in elucidating the complex genetic and molecular basis mediating cold sensitivity, including RP [6, 7]. Secondary RP (SRP) tends to occur in individuals with later-onset (e.g., > 40 years) RP. SRP encompasses broad-ranging underlying medical aetiologies including (but not limited to) systemic autoimmune rheumatic diseases (SARDs), drug causes, and occupational-based exposures [8–10].

Although uncommon, RP occurring in the context of cancer may provide novel epidemiological and mechanistic insights. For example, haematological malignancies may manifest as acral ischaemic syndromes, including SRP (often terminating in critical digital ischaemia). This likely results from increased ‘circulating’ (intravascular) cellular counts and/or plasma viscosity, resulting in occlusive impairment of the microcirculation [9]. In patients with systemic sclerosis (SSc) where RP is almost universally (> 95%) observed [11], anti-RNA polymerase III antibody positivity is strongly associated with the *contemporaneous* clinical onset of SSc and cancer [12]. In this setting, shared (cross-reactive) immune responses against autoantigens target both non-cancerous (i.e., ‘autoimmune’ targets of SSc) and cancerous tissues are postulated to contribute [12]. A key step is likely from the loss of local tolerance mechanism/s and through amplification of anti-tumour immune responses, leading to the generation of systemic autoimmunity [13]. Additionally, antiphospholipid antibodies may contribute to vascular disturbances in SRP associated with malignancy, thereby linking cancer, autoimmunity, and thrombotic pathways.

Against this background, our aim was to describe the risk of cancer in individuals with RP. Our study can be considered as exploratory to inform future research, including examination of cancer-specific risks, and for any important differences seen with age.

## Patients and methods

### Data source

Ours was a retrospective cohort analysis leveraging data from the electronic health records (EHR) of over 140 million individuals across more than 100 healthcare organizations (mostly secondary and tertiary centres in North America), from March 2005 to March 2025; details of which are available from trinetx.com and have been further described elsewhere [14]. For our current study, further ethical approval was not required (and was exempt) because we only used existing de-identified data and did not share or transmit any identifiable data.

Cases with RP were required to have at least two relevant ICD-10 codes (I73.0) and without any known systemic connective tissue diseases (M30–36). We chose to study individuals with irritable bowel syndrome (IBS) as the comparator group for several important reasons. Firstly, its epidemiology is similar to RP (i.e., being common among younger individuals and in female individuals). Secondly, to our knowledge, IBS is not (currently) known to be associated with cancer. We upon decided upon a priori not to compare the patients with RP to ‘healthy’ controls because such individuals are unlikely to have EHR data and/or comparable healthcare contact/utilisation. Comparators with IBS were also required to have two relevant ICD-10 codes (K58), without SARDs (i.e., autoimmune connective tissue diseases) or any colitis (K50–52).

### Covariates

We included the following covariates assessed within one year prior to the index date (i.e., date of the first RP or IBS code respectively in each group): demographics (i.e., age, sex, ethnicity); comorbidities including prior cancers, cardiovascular disease, proxies for smoking (COPD, tobacco use, nicotine dependence), alcohol-related disorders, overweight/obesity, hypertension, dyslipidaemia, type 2 diabetes; and BMI (< 25, 20–30, > 30 kg/m2, or missing). Individuals were required to have at least one year of data prior to the index date; thus, there were no missing data for binary covariates. All codes are provided in Supplementary Table S1.

### Statistical analysis

We stratified all our analyses based upon a priori by age, < or ≥ 45 years. The reasoning for this pragmatic cut-off was two-fold. In particular, we were mindful that certain cancers are more common in particular age groups. Furthermore, we can be much more confident that RP is secondary when symptoms develop after the age of 40 years [4]. We described the unadjusted incidence rate of each outcome and the incidence rate ratio between two comparator groups using Poisson approximation for confidence intervals. To account for potential confounding variables, we compared the risk of each cancer outcome between two exposure groups using 1:1 propensity score-matched Cox proportional hazard models. We took a standardised mean difference (SMD) of less than 0.1 as evidence of satisfactory matching. Our period of follow-up started from one day after the indexed date (the rationale being to reduce the possibility of reverse causation) until the outcome of interest, the date of their last health record entry (e.g., lost to follow-up, emigration, or death), completion of 10 years of follow-up, and/or end of the data cut (14th March 2025).

## Results

Baseline characteristics before propensity score matching are presented in Table 1. Post-matching demographic details between groups are presented in Table 2.

**Table 1:** Unadjusted pre matching baseline characteristics

	< 45 years			≥ 45 years		
	RP	IBS	SMD	RP	IBS	SMD
*N*	35,887	174,647		70,404	284,163	
Age	32.4 (7.9)	31.5 (7.7)	0.117	61.4 (10.6)	61.1 (10.5)	0.026
Female sex	27,498 (79.5)	122,733 (72.8)	0.156	47,563 (69.1)	200,235 (72)	0.065
White	27,207 (78.6)	117,668 (69.8)	0.203	55,169 (80.1)	195,615 (70.4)	0.227
Black or African American	1448 (4.2)	11,628 (6.9)	0.119	2535 (3.7)	16,635 (6)	0.108
Asian	669 (1.9)	6533 (3.9)	0.116	1131 (1.6)	8753 (3.1)	0.099
Cancers	4353 (12.6)	15,320 (9.1)	0.112	17,146 (24.9)	59,171 (21.3)	0.086
Hypertension	2584 (7.5)	9841 (5.8)	0.065	1287 (1.9)	2922 (1.1)	0.068
Ischemic heart diseases	324 (0.9)	955 (0.6)	0.043	6865 (10)	20,846 (7.5)	0.088
Cerebrovascular diseases	320 (0.9)	779 (0.5)	0.056	3596 (5.2)	10,477 (3.8)	0.07
COPD	157 (0.5)	645 (0.4)	0.011	3721 (5.4)	12,373 (4.5)	0.044
Overweight and obesity	2308 (6.7)	14,315 (8.5)	0.069	5770 (8.4)	29,452 (10.6)	0.076
Type 2 diabetes	605 (1.7)	3664 (2.2)	0.031	5238 (7.6)	30,072 (10.8)	0.111
Dyslipidaemia	2131 (6.2)	9799 (5.8)	0.015	21,098 (30.6)	76,124 (27.4)	0.072
Tobacco use	495 (1.4)	1981 (1.2)	0.023	1591 (2.3)	4378 (1.6)	0.053
Nicotine dependence	2088 (6)	9362 (5.6)	0.021	21,788 (31.6)	79,367 (28.5)	0.067
Alcohol-related disorders	564 (1.6)	1877 (1.1)	0.044	1493 (2.2)	3362 (1.2)	0.074
BMI	25.6 (6.4)	28.3 (7.8)	0.381	26.3 (6.2)	29.1 (7.1)	0.424

Data are shown as mean (SD) or *n* (%). *BMI*, body mass index; *COPD*, chronic obstructive pulmonary disease; *IBS*, irritable bowel syndrome; *RP*, Raynaud’s phenomenon; *SMD*, standardised mean difference

**Table 2:** Baseline characteristics after propensity score matching

	< 45 years			≥ 45 years		
	RP	IBS	SMD	RP	IBS	SMD
*N*	34,582	34,582		68,836	68,836	
Age	32.4 (7.9)	32.4 (7.9)	0.002	61.4 (10.6)	61.3 (10.6)	0.005
Female sex	27,484 (79.5)	27,630 (79.9)	0.01	47,556 (69.1)	48,169 (70)	0.019
White	27,193 (78.6)	27,286 (78.9)	0.007	55,147 (80.1)	55,563 (80.7)	0.015
Black or African American	1448 (4.2)	1438 (4.2)	0.001	2535 (3.7)	2510 (3.6)	0.002
Asian	669 (1.9)	673 (1.9)	0.001	1131 (1.6)	1123 (1.6)	0.001
Cancers	4344 (12.6)	4355 (12.6)	0.001	17,131 (24.9)	17,182 (25)	0.002
Hypertension	2572 (7.4)	2450 (7.1)	0.014	1286 (1.9)	778 (1.1)	0.061
Ischemic heart diseases	313 (0.9)	277 (0.8)	0.011	6843 (9.9)	6598 (9.6)	0.012
Cerebrovascular diseases	309 (0.9)	261 (0.8)	0.015	3577 (5.2)	3386 (4.9)	0.013
COPD	156 (0.5)	106 (0.3)	0.024	3707 (5.4)	3438 (5)	0.018
Overweight and obesity	2308 (6.7)	2261 (6.5)	0.005	5770 (8.4)	5612 (8.2)	0.008
Type 2 diabetes	605 (1.7)	515 (1.5)	0.021	5237 (7.6)	4965 (7.2)	0.015
Dyslipidaemia	2125 (6.1)	2074 (6)	0.006	21,081 (30.6)	20,911 (30.4)	0.005
Tobacco use	491 (1.4)	446 (1.3)	0.011	1579 (2.3)	1334 (1.9)	0.025
Nicotine dependence	2083 (6)	1972 (5.7)	0.014	21,764 (31.6)	21,388 (31.1)	0.012
Alcohol-related disorders	560 (1.6)	506 (1.5)	0.013	1476 (2.1)	1288 (1.9)	0.019
BMI	25.6 (6.4)	26.1 (6.8)	0.079	26.3 (6.2)	26.7 (6.4)	0.067

Data are shown as mean (SD) or *n* (%). *BMI*, body mass index; *COPD*, chronic obstructive pulmonary disease; *IBS*, irritable bowel syndrome; *RP*, Raynaud’s phenomenon; *SMD*, standardised mean difference

### Cancer risk under 45 years

We identified 35,887 individuals meeting the case definition for RP and 174,647 matched IBS controls. Our analyses included 34,582 matched pairs, followed over a mean of 4.4 (SD 3.3) and 4.5 (SD 3.2) years, respectively. For RP and IBS groups, respectively, cancer events occurred in 1457 and 1343 individuals, over 152,262 and 155,624 patient-years. The incidence of all cancer [IRR 1.11 (1.03, 1.19)] was higher in the RP group than the IBS group. The hazard of all cancer [HR 1.107 (1.028, 1.192)] was similarly higher in the RP group. Incidence rates of cancer in the matched populations are presented in Table 3. The incidence and hazards of certain cancers were higher in the RP compared to the IBS group: thorax [2.09 (1.28, 3.40) & 2.077 (1.274 & 3.385)], skin [1.20 (1.03, 1.41) & 1.202 (1.028, 1.405)], haematological [1.65 (1.37, 2.00) & 1.647 (1.362, 1.992)], and male genital [4.28 (2.48, 7.38) & 4.257 (2.467, 7.344)]. This was also observed for head and neck [1.069 (0.57, 2.02) & 1.069 (0.571, 2.003)] and female genital [1.12 (0.88, 1.43) & 1.118 (0.875 & 1.429)], although the estimates lacked precision. There was a reduced incidence and hazard of digestive cancer [0.69 (0.52, 0.91) & 0.686 (0.519, 0.906)], and similarly, for breast cancer [0.86 (0.73, 1.01) & 0.853 (0.724, 1.005)], although the latter estimate lacked precision.

**Table 3:** Incidence rates in propensity matched (< 45 and ≥ 45 years) populations

Outcome	RP Events	RP PYs	RP IR/1000PY (95% CI)	IBS Events	IBS PYs	IBS IR/1000PY (95% CI)	IRR (95% CI)	HR (95% CI)
** < 45 years**								
Any cancer	1457	152,262	9.6 (8.9, 10.3)	1343	155,624	8.6 (8.0, 9.3)	1.11 (1.03, 1.19)	1.107 (1.028, 1.192)
Head and neck	20	152,262	0.1 (0.1, 0.2)	19	155,624	0.1 (0.1, 0.2)	1.08 (0.57, 2.02)	1.069 (0.571, 2.003)
Digestive organ	83	152,262	0.5 (0.4, 0.7)	123	155,624	0.8 (0.6, 1.0)	0.69 (0.52, 0.91)	0.686 (0.519, 0.906)
Thorax	49	152,262	0.3 (0.2, 0.5)	24	155,624	0.2 (0.1, 0.3)	2.09 (1.28, 3.40)	2.077 (1.274, 3.385)
Skin	344	152,262	2.3 (1.9, 2.6)	292	155,624	1.9 (1.6, 2.2)	1.20 (1.03, 1.41)	1.202 (1.028, 1.405)
Breast	263	152,262	1.7 (1.5, 2.0)	313	155,624	2.0 (1.7, 2.4)	0.86 (0.73, 1.01)	0.853 (0.724, 1.005)
Haematological	278	152,262	1.8 (1.5, 2.2)	172	155,624	1.1 (0.9, 1.3)	1.65 (1.37, 2.00)	1.647 (1.362, 1.992)
Male genital	67	152,262	0.4 (0.3, 0.8)	16	155,624	0.1 (0.1, 0.2)	4.28 (2.48, 7.38)	4.257 (2.467, 7.344)
Female genital	134	152,262	0.9 (0.7, 1.1)	122	155,624	0.8 (0.6, 1.0)	1.12 (0.88, 1.43)	1.118 (0.875, 1.429)
** ≥ 45 years**								
Any cancer	13,683	333,238	41.1 (40.1, 42.1)	13,002	342,725	37.9 (37.0, 38.9)	1.08 (1.06, 1.11)	1.08 (1.054, 1.106)
Head and neck	380	333,238	1.1 (1.0, 1.3)	248	342,725	0.7 (0.6, 0.8)	1.58 (1.34, 1.85)	1.563 (1.332, 1.834)
Digestive organ	1406	333,238	4.2 (3.9, 4.5)	1600	342,725	4.7 (4.3, 5.0)	0.90 (0.84, 0.97)	0.894 (0.832, 0.960)
Thorax	1487	333,238	4.5 (4.1, 4.8)	1061	342,725	3.1 (2.9, 3.3)	1.44 (1.33, 1.56)	1.433 (1.325, 1.551)
Skin	4540	333,238	13.6 (13.1, 14.2)	3868	342,725	11.3 (10.8, 11.8)	1.21 (1.16, 1.26)	1.213 (1.162, 1.266)
Breast	2629	333,238	7.9 (7.5, 8.3)	2908	342,725	8.5 (8.0, 8.9)	0.93 (0.88, 0.98)	0.919 (0.872, 0.969)
Haematological	1788	333,238	5.4 (5.0, 5.8)	1364	342,725	4.0 (3.7, 4.3)	1.35 (1.26, 1.45)	1.338 (1.247, 1.436)
Male genital	1348	333,238	4.0 (3.7, 4.4)	1287	342,725	3.8 (3.5, 4.1)	1.08 (1.00, 1.16)	1.068 (0.989, 1.152)
Female genital	828	333,238	2.5 (2.3, 2.7)	927	342,725	2.7 (2.5, 3.0)	0.92 (0.84, 1.01)	0.909 (0.828, 0.998)

*RP*, Raynaud’s phenomenon; *HR*, hazard ratio; *IBS*, irritable bowel syndrome; *IRR*, incidence rate ratio; *PY*, patient year

### Cancer risk over 45 years

We identified 70,404 individuals meeting the case definition for RP and 284,163 matched IBS controls. Our analyses included 68,836 matched pairs, followed over a mean of 4.8 (SD 3.3) and 5.0 (SD3.2) years, respectively. For RP and IBS groups, respectively, cancer events occurred in 13,683 and 13,002, over 333,238 and 342,725 patient-years of follow-up. The incidence of all cancer [IRR 1.08 (1.06, 1.11)] was higher in the RP group than in the IBS group. The hazard of all cancer [HR 1.08 (1.054, 1.106)] was similarly higher in the RP group. Incidence rates of cancer in the matched populations are presented in Table 3. The incidence and hazards of certain cancers were higher in the RP compared to the IBS group: head and neck [1.58 (1.34, 1.85) & 1.563 (1.332, 1.834)], thorax [1.44 (1.33, 1.56) & 1.433 (1.325, 1.551)], skin [1.21 (1.16, 1.26) & 1.213 (1.162, 1.266)], and haematological [1.35 (1.26, 1.45) & 1.338 (1.247, 1.436)]. This was also observed for male genital cancer [1.08 (1.00, 1.16) & 1.068 (0.989, 1.152)], although the estimate lacked precision. There was a reduced incidence and hazard of digestive [0.90 (0.84, 0.97) & 0.894 (0.832, 0.960)] and breast [0.93 (0.88, 0.98) & 0.919 (0.872, 0.969)] cancers.

## Discussion

To our knowledge, ours is the first study to examine cancer risk in individuals with RP. The key finding of our study is that RP was associated with an overall increased risk of cancer in those both under and over 45 years of age. An important strength of our study was that we were able to approximate a cohort of individuals phenotypically closer to PRP, in an attempt to examine the individual impact of RP on cancer risk. Our study benefited from the inclusion of a large number of well-characterised patients and from comparing to a well-matched comparator cohort (of individuals with IBS).

Our study provided the opportunity to generate valuable estimates concerning cancer-specific risks in RP. Independent of age, RP was associated with an increased risk of thorax (HR < 45 2.077, HR ≥ 45 1.433), skin (HR < 45 1.202, HR ≥ 45 1.213), and haematological (HR < 45 1.647, HR ≥ 45 1.338) cancers. Furthermore, an excess risk was also observed, although estimates lacked precision, for head and neck cancer (HR < 45 1.069, HR ≥ 45 1.563). The risk of male genital cancer was significantly elevated in individuals under 45 (HR = 4.257), whereas in those over 45, the increase was less pronounced, and estimates lacked precision (HR = 1.068). Interestingly, a biphasic risk profile was observed with female genital cancer: with an increased risk in younger (HR < 45 1.118) and a reduced risk in older (HR ≥ 45 0.909) individuals. RP was associated with a decreased risk of GI (HR < 45 0.686, HR ≥ 45 0.894) and breast (HR < 45 0.853, HR ≥ 45 0.919) cancers, although the estimates lacked precision in the younger breast cancer group.

Although our study was not able to infer causality between RP and cancer, the findings are of major significance considering that Raynaud’s affects ~ 3–5% of the general population. The scientific basis underpinning an association specifically between RP and cancer currently has not been explored. However, conceptually, RP is characterised by episodes of ischaemia–reperfusion, which could theoretically result in adverse tissue injury promoting a local pro-mitogenic milieu. Furthermore, there could be shared risk factors, including lifestyle behaviours and occupational exposures, that are potentially relevant (even after our extensive propensity matching). In patients with SARDs, including SSc and dermatomyositis, clear aetiopathogenic links with cancer are established, including the role of autoantibodies as biomarkers of cancer in the setting of systemic autoimmunity. Future research could examine for a common genetic architecture between RP and cancer, including with specific cancer types.

A major strength of our study was the large sample size, including the use of a well-matched comparator cohort; however, there are several points warranting discussion. We specifically compared individuals with RP and those with IBS; our rationale being that the epidemiology of IBS is well known and comparable to those phenotypically with PRP. Furthermore, to our knowledge, no significant overall increase in risk, including of GI cancer, has been reported in extant literature [15]. By specifically excluding individuals with RP with known SARDs (akin to PRP), we were able to more closely approximate the risk of cancer which might be attributed to RP. Future studies could also examine the temporal relationship and absolute risk of cancer and Raynaud’s phenomenon. A limitation is that the diagnosis of RP was not subject to further confirmatory clinician chart review, including for the detection of ‘other’ possible causes of SRP. It could also be argued that some individuals could also have had undiagnosed (or early forms) of SARDs; however, there is no reason to expect that this would have significantly differed between groups. Furthermore, RP characteristics, including the nature of the digital colour changes and/or severity, were not available, which could be explored in future studies [16]. Potential misclassification and under-reporting of exposure are possible when using administrative data covariates and outcomes are possible; for example, individuals who use tobacco may not declare it, therefore it will not appear in coded data. However, this is unlikely to differ systematically between exposure groups. Future studies should examine the time between the onset and diagnosis of RP to provide further mechanistic insights into pathobiology. In addition, future studies could also examine conceptually related SARDs with a significant SSc-phenotype (e.g., mixed connective tissue disease) and cold sensitivity syndromes (e.g., acrocyanosis). To highlight, our data warrants further validation and exploration and should not currently be used to inform clinical decision-making. However, future research might look to produce final clinical recommendations for cancer risk assessment in RP, incorporating important patient demographics and exploring the utility of recognising Raynaud’s symptom characteristics (e.g., the relative risk conferred by mono/biphasic vs. triphasic attacks).

In conclusion, in our large population-based cohort study, RP was associated with an overall increased risk of cancer in both younger and older individuals. Our data also highlights an increased risk of certain (e.g., skin, thorax, and haematological) cancers and appeared to be protective for others (e.g., GI and breast). Further research is indicated to confirm and expand upon these observations, including to explore potential insights into shared pathogenic mechanisms.

## Supplementary Information

Below is the link to the electronic supplementary material.

ESM1 (DOCX 15.3 KB)

## Data Availability

The data that support the findings of this study are available from TriNetX, LLC, but third-party restrictions apply to the availability of these data. The data were used under license for this study with restrictions that do not allow for the data to be redistributed or made publicly available. However, for accredited researchers, the TriNetX data is available for licensing at TriNetX, LLC. To gain access to the data in the TriNetX research network, a request can be made to TriNetX (https://live.trinetx.com), but costs may be incurred, a data sharing agreement would be necessary, and no patient identifiable information can be obtained. No data from Liverpool University Hospitals NHS Foundation Trust was utilized in this analysis.

## References

[1] Wigley FM, Flavahan NA (2016) Raynaud’s phenomenon. N Engl J Med 375:556–565 27509103 10.1056/NEJMra1507638

[2] Devgire V, Hughes M (2019) Raynaud’s phenomenon. Br J Hosp Med (Lond) 80:658–664 31707892 10.12968/hmed.2019.80.11.658

[3] Stjernbrandt A, Pettersson H, Lundström R et al (2022) Incidence, remission, and persistence of Raynaud’s phenomenon in the general population of northern Sweden: a prospective study. BMC Rheumatol 6:41 35858907 10.1186/s41927-022-00272-0 PMC9301854

[4] Maundrell A, Proudman SM (2015) Epidemiology of Raynaud’s phenomenon. In: Wigley FM, Herrick AL, Flavahan NA (eds) Raynaud’s phenomenon: from pathogenesis to management, 1st edn. Springer, New York, pp 21–35

[5] Temprano KK (2016) A review of Raynaud’s disease. Mo Med 113:123–126 27311222 PMC6139949

[6] Garner R, Kumari R, Lanyon P et al (2015) Prevalence, risk factors and associations of primary Raynaud’s phenomenon: systematic review and meta-analysis of observational studies. BMJ Open 5:e006389–e006389 25776043 10.1136/bmjopen-2014-006389 PMC4368987

[7] Hartmann S, Yasmeen S, Jacobs BM et al (2023) ADRA2A and IRX1 are putative risk genes for Raynaud’s phenomenon. Nat Commun 14:6156 37828025 10.1038/s41467-023-41876-5 PMC10570309

[8] Pauling JD, Hughes M, Pope JE (2019) Raynaud’s phenomenon - an update on diagnosis, classification and management. Clin Rheumatol 2019:3317–3330 10.1007/s10067-019-04745-5 31420815

[9] Hughes M, Shah AA (2024) Other Secondary Causes. In: Wigley FM, Herrick AL, Flavahan NA (eds) In Raynaud’s phenomenon: from pathogenesis to management, 2nd edition. Springer Nature, Switzerland, pp 141–67

[10] Di Donato S, Huang S, Pauling JD et al (2024) Clinically relevant differences between primary Raynaud’s phenomenon and secondary to connective tissue disease. Semin Arthritis Rheum 68:152521 39089171 10.1016/j.semarthrit.2024.152521

[11] Meier FMP, Frommer KW, Dinser R et al (2012) Update on the profile of the EUSTAR cohort: an analysis of the EULAR Scleroderma Trials and Research group database. Ann Rheum Dis 71:1355–60 22615460 10.1136/annrheumdis-2011-200742

[12] Moinzadeh P, Fonseca C, Hellmich M et al (2014) Association of anti-RNA polymerase III autoantibodies and cancer in scleroderma. Arthritis Res Ther 16:R53 24524733 10.1186/ar4486 PMC3978927

[13] Cappelli LC, Shah AA (2020) The relationships between cancer and autoimmune rheumatic diseases. Best Pract Res Clin Rheumatol 34:101472 32029389 10.1016/j.berh.2019.101472 PMC7295675

[14] Ludwig RJ, Anson M, Zirpel H, Thaci D, Olbrich H, Bieber K, et al (2025) A comprehensive review of methodologies and application to use the real-world data and analytics platform TriNetX. Front Pharmacol 16:1516126 10.3389/fphar.2025.1516126 PMC11931024 40129946

[15] Wu S, Yuan C, Liu S et al (2022) Irritable bowel syndrome and long-term risk of cancer: a prospective cohort study among 0.5 million adults in UK Biobank. Am J Gastroenterol 117:785–93 35130187 10.14309/ajg.0000000000001674

[16] Hughes M, Huang S, Pauling JD, Sabbagh M, Khanna D (2020) The clinical relevance of Raynaud’s phenomenon symptom characteristics in systemic sclerosis. Clin Rheumatol 41:3049–3054 10.1007/s10067-022-06206-y 35583625

