# Supplementary material for: An epidemiological study estimating the burden of cancer risk in patients with Raynaud’s phenomenon
Source: Clin Rheumatol. 2026 Jan 29;45(3):1651–6. doi: 10.1007/s10067-026-07961-y (PMC12923405; doi:10.1007/s10067-026-07961-y)
Supplement: Supplementary file 1 — (DOCX 15.3 KB) [file 10067_2026_7961_MOESM1_ESM.docx]

Supplementary Table S1. ICD codes used to define diseases and covariates.

| **Trait** | **ICD‑10 code(s)** |
| --- | --- |
| Raynaud’s phenomenon | I73.0 without M30-36 |
| Irritable bowel syndrome | K58 without M30-36 or K50-52 |
| Hypertension | I10 |
| Ischemic heart diseases | I20 – I25 |
| Cerebrovascular diseases | I60 – I69 |
| COPD | J44 |
| Overweight and obesity | E66 |
| Type 2 diabetes | E11 |
| Dyslipidaemia | E78 |
| Tobacco use | Z72.0 |
| Nicotine dependence | F17 |
| Alcohol‑related disorders | F10 |
| Any cancer | C00 – D49 |
| Head & neck cancer | C00 – C14 |
| Digestive‑organ cancer | C15 – C26 |
| Thorax (respiratory/intrathoracic) cancer | C30 – C39 |
| Skin cancer | C43 – C44 |
| Breast cancer | C50 |
| Haematological (lymphoid/haematopoietic) cancer | C81 – C96 |
| Male‑genital cancer | C60 – C63 |
| Female‑genital cancer | C51 – C58 |
